# 2084. Importance of the Primary Care Physician in HIV PrEP Adherence During a Pandemic

**DOI:** 10.1093/ofid/ofac492.1706

**Published:** 2022-12-15

**Authors:** Isaac Daudelin, Bret McCarty, Shawn K Ahmad Chaustre, Diana Finkel

**Affiliations:** Rutgers NJMS, Union, New Jersey; Rutgers NJMS, Union, New Jersey; Rutgers NJMS, Union, New Jersey; Rutgers NJMS, Union, New Jersey

## Abstract

**Background:**

In 2015 emtricitabine-tenofovir (PrEP) was approved for use as a pre-exposure prophylactic medication for preventing HIV infections. In 2017, WHO declared PrEP to be an essential medicine, gaining widespread acceptance for outpatient care. In 2020, the COVID-19 pandemic disrupted the healthcare system, with several studies showing lower rates of PrEP prescriptions during the pandemic particularly among Black and Latinx groups.

**Methods:**

Medical records at Rutgers New Jersey Medical School (NJMS) were queried for PrEP prescriptions from 2017-2021. All duplicate entries, and all patients prescribed emtricitabine and tenofovir as part of HIV treatment were removed. Years 2017-2019 were defined as “pre-pandemic” and years 2020-2021 were defined as “pandemic.”

**Results:**

Average annual prescriptions for PrEP at NJMS decreased by only 13% (157 to 137 p=0.53) for all ages and 25% (29 to 22 p=0.38) for adolescents and young adults (ages 16-24) (AYA) from pre-pandemic to pandemic years. The annual number of AYAs who had their PrEP prescribed by the Division of Adolescent and Young Adult Medicine practice (DAYAM) remained the same during the pandemic while the annual number of AYAs who had their PrEP prescribed by the Infectious Disease practice (IDP) decreased by over 50% (p = 0.07). PrEP prescriptions for trans-identifying individuals (over 90% prescribed by the IDP) increased by 15% (p = 0.58).

PrEP Prescriptions for Adolescents and Young Adults

**Figure:**
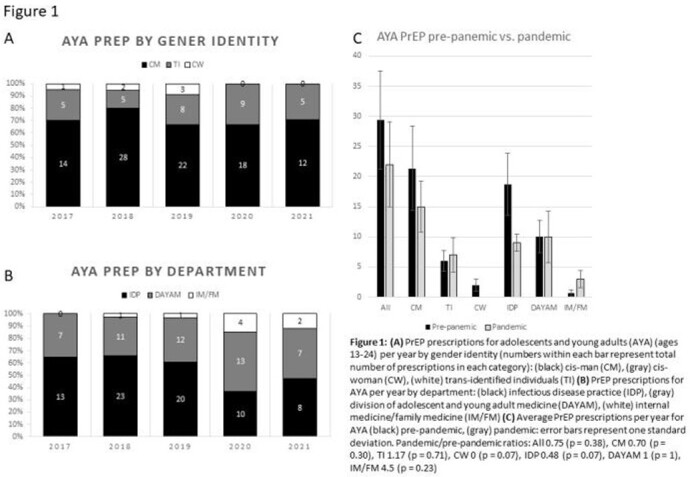

All PrEP Prescriptions at Rutgers NJMS

**Figure:**
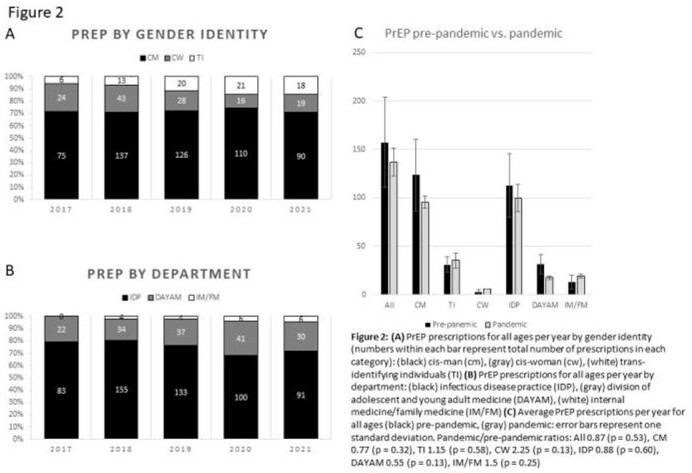

**Conclusion:**

Despite the national decline in PrEP prescriptions during the COVID-19 pandemic, NJMS experienced a non-statistically significant decrease in PrEP prescriptions despite serving a majority Black and Latinx population who have elsewhere suffered the most disruption in healthcare during the pandemic. At NJMS, the IDP provides primary care for trans-identifying individuals, and DAYAM provides primary care for AYAs. Average annual PrEP prescriptions for trans-identifying individuals increased and AYA PrEP prescriptions by the DAYAM remained the same throughout the pandemic emphasizing the key role primary care physicians have in PrEP access during the pandemic and may play a significant role in the strong continuation of PrEP program provided at NJMS throughout the pandemic.

**Disclosures:**

**All Authors**: No reported disclosures.

